# Rituximab or a second anti–tumor necrosis factor therapy for rheumatoid arthritis patients who have failed their first anti–tumor necrosis factor therapy? Comparative analysis from the British Society for Rheumatology Biologics Register

**DOI:** 10.1002/acr.21663

**Published:** 2012-08

**Authors:** Moetaza M Soliman, Kimme L Hyrich, Mark Lunt, Kath D Watson, Deborah P M Symmons, Darren M Ashcroft

**Affiliations:** University of ManchesterManchester, UK

## Abstract

**Objective:**

To compare the effectiveness of rituximab (RTX) or a second anti–tumor necrosis factor (anti-TNF) therapy in rheumatoid arthritis (RA) patients who had failed their first anti-TNF and switched to either RTX or a second anti-TNF, in routine clinical practice.

**Methods:**

RA patients were registered with the British Society for Rheumatology Biologics Register. Response to treatment 6 months after switching was assessed using European League Against Rheumatism (EULAR) criteria and improvements in a Health Assessment Questionnaire (HAQ) score (0.22 unit or more). Regression analyses were used to compare EULAR response and improvement in HAQ score between the 2 groups, adjusting for propensity scores.

**Results:**

In total, 1,328 patients were included in the analysis of EULAR response, and 937 patients were included in the analysis of HAQ scores. Six months after switching, 54.8% of patients who switched to RTX were EULAR responders compared to 47.3% of those who switched to a second anti-TNF. A total of 38.4% of RTX patients achieved a clinically important improvement in HAQ score compared to 29.6% in anti-TNF patients. After adjustment using propensity scores, patients who switched to RTX were significantly more likely to achieve EULAR response (odds ratio [OR] 1.31; 95% confidence interval [95% CI] 1.02, 1.69) compared to those who switched to an alternative anti-TNF. RTX patients were also significantly more likely to achieve improvements in HAQ score (OR 1.49; 95% CI 1.07, 2.08).

**Conclusion:**

The results suggest that switching to RTX may be of more benefit than switching to an alternative anti-TNF therapy after failing the first anti-TNF therapy in RA patients.

## INTRODUCTION

In recent years, anti–tumor necrosis factor (anti-TNF) therapies have been routinely used for the management of rheumatoid arthritis (RA) patients who have failed traditional nonbiologic disease-modifying antirheumatic drugs (DMARDs). However, approximately 30% of the patients discontinue treatment with anti-TNF therapy within 1 year due either to inefficacy or adverse events (1). In those patients who had failed their initial anti-TNF therapy, studies have shown that switching to a second alternative anti-TNF therapy can be effective (2–5).

The British Society for Rheumatology receives restricted income from UK pharmaceutical companies, presently Abbott Laboratories, Amgen, Roche, Schering-Plough, and Wyeth Pharmaceuticals. Dr. Soliman's work was supported by the Egyptian Government.

Moetaza M. Soliman, MSc, PhD, Kimme L. Hyrich, MD, PhD, FRCPC, Mark Lunt, PhD, Kath D. Watson, PhD, Deborah P. M. Symmons, MD, FFPH, FRCP, Darren M. Ashcroft, BPharm, MSc, PhD: University of Manchester, Manchester, UK.

Rituximab (RTX), a chimeric monoclonal antibody that acts by depleting B cells, was introduced in 2006 for the management of RA patients who have failed 1 or more anti-TNF therapies. RTX has been shown to be effective in both clinical trials (6–8) and observational studies (9–11).

After RTX was introduced, patients who have failed anti-TNF therapy may either switch to an alternative anti-TNF or use RTX. Consequently, an important clinical question is raised. Which treatment option is more effective? There are no published randomized clinical trials that have compared RTX to an alternative anti-TNF therapy directly. An earlier prospective cohort study of 116 patients has suggested that RTX may be more effective in terms of a change in the Disease Activity Score in 28 joints (DAS28) and inflammation markers (12). To date, there are no comparative studies that have reported on improvements in physical function.

Therefore, the current analysis aimed to compare the effectiveness of RTX versus a second anti-TNF therapy in RA patients who had failed their first anti-TNF therapy in routine clinical practice. The measures of effectiveness included both improvement in clinical outcomes (European League Against Rheumatism [EULAR] criteria) and patient-reported physical function (improvements in the Health Assessment Questionnaire [HAQ] score).

**Significance & Innovations**

- Switching to rituximab (RTX) was found to be more effective than switching to a second alternative anti–tumor necrosis factor (anti-TNF) therapy after failing a first anti-TNF therapy.
- Patients who switched to RTX were significantly more likely to achieve a European League Against Rheumatism response.
- Patients who switched to RTX were significantly more likely to achieve improvements in physical function.

## PATIENTS AND METHODS

### Patient population

The current analysis used patients who were registered with the British Society for Rheumatology Biologics Register (BSRBR) (13). The BSRBR is a national prospective observational study recruiting RA patients who receive biologic therapies in the UK. Recruitment to the anti-TNF cohorts started in 2001 and to the RTX cohort in 2008. Registration to the RTX cohort was open for both patients who were previously in the register as anti-TNF patients and switched to RTX (and subsequently reregistered at the time RTX was started) and patients who have never been in the register before. In both cases, the patient should have started RTX within 6 months prior to registration. Patients who had received their first dose of RTX >6 months prior to the opening of the formal RTX cohort remained in their original anti-TNF cohort only for ongoing followup. The sample size target (4,000 for each of the original 3 anti-TNF agents and 1,100 for RTX patients) was calculated based on the power to detect a doubling in risk of lymphoma compared to standard nonbiologic DMARD therapies for the 3 anti-TNF therapies and on the power to detect a doubling of the risk of serious infection in the case of RTX. The target for recruitment of anti-TNF patients and RTX patients was achieved in 2008 and 2011, respectively.

### Ethical approval

Ethical approval for the BSRBR was granted by the North West Multi-Centre Research Ethics Committee in December 2000. The approval was then extended in January 2007 to recruit patients who have been treated with RTX. All patients provided written informed consent.

### Baseline data and followup

At the time of patient registration with the BSRBR, a consultant baseline questionnaire was completed by the consultant rheumatologist. The questionnaire collected data on patient demographics and disease characteristics, including the DAS28 score. For patients who were reregistered with the BSRBR when they switched to RTX, the DAS28 score was collected at the time of starting RTX. For RTX patients who were not previously in the register, the previous biologic history and the reason for discontinuation were collected at the time of registration. All the patients were asked to complete the HAQ, adapted for use in a UK population, at the start of the therapy (14).

The BSRBR aimed to follow up with all the patients at 6-month intervals for 3 years and then annually thereafter, even if the patient stopped or switched their therapy. Consultants were asked to complete followup questionnaires that collected data on any changes to biologic therapy, as well as the DAS28 score along with its date. After baseline, DAS28 and HAQ scores were not collected specifically at times of drug starts and stops. If the patient stopped therapy, start and stop dates and the reason for discontinuation were documented. Patients also completed the HAQ at 6-month intervals.

### Inclusion and exclusion criteria

BSRBR data, up to December 21, 2010, were used for the current comparative analysis. All RA patients registered with the BSRBR, who had failed their first anti-TNF therapy for any reason and then switched to either RTX or a second alternative anti-TNF therapy, were eligible for inclusion in this analysis. If a patient switched to an alternative anti-TNF therapy on more than one occasion, then the first switch would be the only kept switch for that patient. To be included in the analysis, the patient needed to have DAS28 and/or HAQ scores recorded within 3 months before switching and at 6 (±3) months after switching.

### Statistical analysis

As not all patients had both a HAQ and DAS28 score recorded at drug start and at 6-months posttreatment, we performed 2 separate parallel analyses to maximize the statistical power. The first included all patients with a DAS28 score at drug start and at 6-months posttreatment. The primary outcome was achieving a EULAR response (15) with secondary outcomes of the change in DAS28 score (16), and the proportions of patients achieving remission (DAS28 <2.6) according to the EULAR criteria (17).

The second analysis included all patients with a HAQ score recorded at the start of treatment and 6-months posttreatment. The primary outcome was achieving a minimum clinically important difference (MCID) on the HAQ (18) 6 months after switching with a secondary outcome of the change in HAQ score. In both analyses, the response was compared between the patients who switched to RTX and the patients who switched to a second alternative anti-TNF therapy. Patients who switched their therapy again (or stopped it) within the 6 months of followup were categorized as EULAR nonresponders and as not achieving a MCID for the HAQ.

Given that patients were treated in routine clinical practice and therefore were not randomized to the therapy they switched to, it was essential to adjust for differences between the 2 groups of patients that may affect their response to the new therapy, such as baseline disease severity. Propensity scores were calculated using logistic regression models and were used to adjust for the differences in baseline characteristics of the 2 groups of patients (19). Two separate propensity score models were developed, one for patients with EULAR data and the other for patients with HAQ data. Since each propensity score model included different patient populations, each model included different variables to adjust for any differences in baseline characteristics of the included patients. For patients with EULAR data, the propensity score included DAS28 score, comorbidities, the failed anti-TNF therapy, and interaction between age (at time of switch), and the reason for switching. For patients with HAQ data, the propensity score included age (at time of switch), comorbidities, the last failed anti-TNF therapy, interaction between disease duration (at time of switch), and the reason for switching.

Ordinal regression models were used to compare the EULAR response rates in the 2 groups of patients 6 months after switching. Logistic regression models were used in both the case of classification of the patients as responders or nonresponders according to the EULAR response and achieving a MCID (at least 0.22-unit changes) in the HAQ score. In all cases, unadjusted and adjusted models (adjusted for propensity score) were calculated. The results are presented as odds ratios (ORs) with the 95% confidence intervals (95% CIs). Stata software, version 10.1, was used to undertake all statistical analyses.

## RESULTS

### Patients

**Figure 1:**
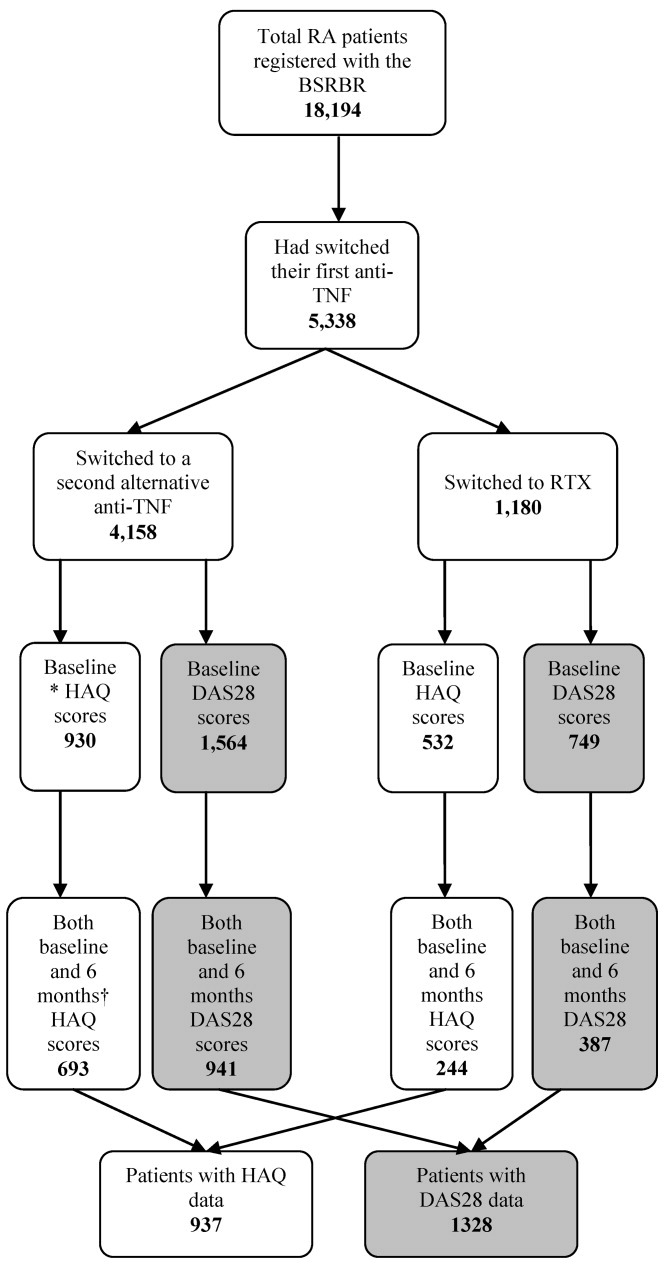
Flow diagram of rheumatoid arthritis (RA) patients who switched to either rituximab (RTX) or an alternative anti–tumor necrosis factor (anti-TNF) therapy after failing their first anti-TNF therapy. BSRBR = British Society for Rheumatology Biologics Register; HAQ = Health Assessment Questionnaire; DAS28 = Disease Activity Score in 28 joints; * = baseline data correspond to data within 3 months before switching; † = 6-month data correspond to data within 3–9 months after switching.

By December 21, 2010, a total of 5,338 patients had switched their first anti-TNF therapy, and 4,158 of those patients then switched to a second alternative anti-TNF therapy and 1,180 switched to RTX (Figure 1). A total of 1,328 patients had DAS28 scores reported at both baseline (time of switching) and at 6 months after switching. A total of 937 patients had HAQ scores reported at baseline and at 6 months.

### Baseline characteristics

Baseline characteristics (at time of switching) of the patients with DAS28 data are shown in Table 1. A total of 387 patients switched to RTX and 941 patients switched to a second alternative anti-TNF therapy. Some differences in the baseline characteristics were observed between the 2 groups of patients. Patients who switched to RTX were generally older, and a larger proportion had comorbidities. RTX patients also had a higher mean ± SD DAS28 score (6.2 ± 1.2 compared to 5.9 ± 1.4 in the anti-TNF patients). Inefficacy of the discontinued anti-TNF was more commonly associated with patients who switched to a second anti-TNF (58.1%) than in those who switched to RTX (49.1%).

**Table 1:** Baseline characteristics at time of switching for patients with DAS28 scores*

Characteristics	Switched to RTX	Switched to anti-TNF	*P*†
Patients, no.	387	941	–
Age, mean ± SD years	58.7 ± 11.2	55.6 ± 12.3	< 0.0001
Female sex, no. (%)	300 (77.5)	757 (80.5)	0.23
Disease duration, mean ± SD years	14.7 ± 10.2	14.0 ± 9.5	0.25
RF positive, no. (%)	242 (64.9)	604 (64.2)	0.06
Comorbidities, no. (%)‡	259 (66.9)	546 (58.0)	0.003
TJC, mean ± SD no.	14.4 ± 8.1	12.5 ± 8.3	< 0.001
SJC, mean ± SD no.	8.7 ± 5.8	8.2 ± 6.1	0.17
ESR, mean ± SD mm/hour	45.0 ± 30.5	43.0 ± 28.5	0.30
CRP level, mean ± SD mg/dl	33.6 ± 35.0	39.7 ± 46.0	0.11
Global health VAS score, mean ± SD	68.5 ± 20.8	63.6 ± 25.1	< 0.001
DAS28, mean ± SD	6.2 ± 1.2	5.9 ± 1.4	< 0.001
Reason for stopping first anti-TNF			0.001
Inefficacy, no. (%)	190 (49.1)	547 (58.1)	
Adverse events, no. (%)	96 (24.8)	257 (27.3)	
Other/missing, no. (%)	101 (26.1)	137 (14.6)	
First anti-TNF therapy			
Etanercept, no. (%)	159 (41.1)	266 (28.3)	< 0.001
Monoclonal antibody, no. (%)	228 (58.9)	675 (71.7)	
Therapy switched to, no. (%)			
Etanercept	–	494 (52.5)	
Infliximab	–	102 (11.0)	
Adalimumab	–	344 (36.5)	

* DAS28 = Disease Activity Score in 28 joints; RTX = rituximab; anti-TNF = anti–tumor necrosis factor; RF = rheumatoid factor; TJC = tender joint count; SJC = swollen joint count; ESR = erythrocyte sedimentation rate; CRP = C-reactive protein; VAS = visual analog scale.

† Test of significance between patients switched to RTX or a second alternative anti-TNF therapy.

‡ Comorbidities included one or more of angina, hypertension, myocardial infarction, stroke, epilepsy, asthma, chronic obstructive pulmonary disease, peptic ulcer, liver disease, renal disorder, demyelination, diabetes mellitus, hyperthyroidism, depression, or history of tuberculosis and cancer.

The baseline characteristics of the patients with HAQ data are shown in Table 2. A total of 244 patients switched to RTX and 693 patients switched to a second alternative anti-TNF therapy. The patients who switched to RTX were generally older and had more comorbidity. The mean baseline HAQ scores were similar between the 2 groups of patients.

**Table 2:** Baseline characteristics at time of switching for patients with HAQ scores*

Characteristics	Switched to RTX	Switched to anti-TNF	*P*†
Patients, no.	244	693	–
Age, mean ± SD years	60.2 ± 10.8	57.7 ± 11.4	< 0.01
Female sex, no. (%)	189 (77.5)	558 (80.5)	0.31
Disease duration, mean ± SD years	15.9 ± 11.1	14.6 ± 9.2	0.08
RF positive, no. (%)	166 (68.0)	444 (64.1)	0.15
Comorbidities, no. (%)‡	166 (68.0)	420 (60.6)	0.04
HAQ, mean ± SD	1.99 ± 0.61	1.96 ± 0.58	0.49
Reason for stopping first anti-TNF, no. (%)			0.007
Inefficacy	113 (46.5)	383 (55.2)	
Adverse events	75 (30.6)	208 (30.1)	
Other/missing	56 (22.9)	102 (14.7)	
First anti-TNF therapy, no. (%)			< 0.001
Etanercept	103 (42.2)	185 (26.7)	
Monoclonal antibody	141 (57.8)	508 (73.3)	
Therapy switched to, no. (%)			
Etanercept	–	380 (54.8)	
Infliximab	–	58 (8.4)	
Adalimumab	–	255 (36.8)	

* HAQ = Health Assessment Questionnaire; RTX = rituximab; anti-TNF = anti–tumor necrosis factor; RF = rheumatoid factor.

† Test of significance between patients switched to RTX or a second alternative anti-TNF therapy.

‡ Comorbidities included one or more of angina, hypertension, myocardial infarction, stroke, epilepsy, asthma, chronic obstructive pulmonary disease, peptic ulcer, liver disease, renal disorder, demyelination, diabetes mellitus, hyperthyroidism, depression, or history of tuberculosis and cancer.

### Disease activity

The mean (95% CI) improvements in the DAS28 scores were similar among the patients who switched to RTX and those who switched to a second alternative anti-TNF therapy (*P* = 0.12). The change in DAS28 was −1.3 (−1.5, −1.2) in patients who switched to RTX compared to −1.2 (−1.3, −1.1) in those who switched to an alternative anti-TNF agent (Table 3). Within the 6 months of followup, 283 patients had either stopped their therapy or switched again and therefore were classified as EULAR nonresponders.

**Table 3:** Six-months response in disease activity measures and patient-reported physical function*

Outcomes	Switched to RTX	Switched to anti-TNF	*P*†
Disease activity measures			
Patients, no.	387	941	
Baseline DAS28 score	6.2 (6.1, 6.3)	5.9 (5.8, 6.0)	< 0.001
Six-months DAS28 score	4.9 (4.7, 5.0)	4.7 (4.6, 4.8)	0.15
Change in DAS28 score	−1.3 (−1.5, −1.2)	−1.2 (−1.3, −1.1)	0.12
EULAR response, no. (%)			
Good	66 (17.1)	127 (13.5)	0.04
Moderate	146 (37.7)	318 (33.8)	
None	175 (45.2)	496 (52.7)	
Achieving remission	28 (7.24)	98 (10.4)	0.07
Physical function outcomes			
Patients, no.	244	693	–
Baseline HAQ score	1.99 (1.91, 2.07)	1.96 (1.92, 2.00)	0.49
Six-months HAQ score	1.86 (1.78, 1.95)	1.85 (1.80, 1.90)	0.81
Change in HAQ score	−0.13 (−0.17, −0.08)	−0.11 (−0.13, −0.08)	0.51
Patients achieving 0.22-unit improvement in HAQ score, no. (%)	94 (38.4)	205 (29.6)	0.01

* Values are the mean (95% confidence interval) unless otherwise indicated. RTX = rituximab; anti-TNF = anti–tumor necrosis factor; DAS28 = Disease Activity Score in 28 joints; EULAR = European League Against Rheumatism; HAQ = Health Assessment Questionnaire.

† Test of significance between patients switched to RTX or a second alternative anti-TNF therapy.

Patients who switched to RTX showed better EULAR response rates compared to those who switched to a second alternative anti-TNF therapy (*P* = 0.04). Of the patients who switched to RTX, 17.1% were good responders, 37.7% were moderate responders, and 45.2% were nonresponders compared to the 13.5%, 33.8%, and 52.7%, respectively, of the patients who switched to a second anti-TNF therapy. Disease remission was achieved in 10.4% of the patients who switched to a second anti-TNF therapy and in 7.2% of the RTX patients (*P* = 0.07).

After adjustment for propensity scores, the patients who switched to RTX were significantly more likely to achieve moderate/good EULAR response (OR 1.31 [95% CI 1.02, 1.69]) compared to those who switched to an alternative anti-TNF therapy (Table 4).

**Table 4:** Adjusted and unadjusted regression of 6-months EULAR response and patient-reported physical function*

	Logistic regression of EULAR responders vs. nonresponders				Ordinal regression of EULAR response				Logistic regression of achieving 0.22-unit improvement in HAQ score			
Therapy	Unadjusted	*P*	Adjusted†	*P*	Unadjusted	*P*	Adjusted†	*P*	Unadjusted	*P*	Adjusted‡	*P*
Anti-TNF (reference)												
RTX	1.35 (1.06, 1.71)	0.01	1.31 (1.02, 1.69)	0.04	1.34 (1.07, 1.68)	0.01	1.34 (1.05, 1.70)	0.02	1.48 (1.09, 2.01)	0.01	1.49 (1.07, 2.08)	0.02

* Values are the odds ratio (95% confidence interval) unless indicated otherwise. EULAR = European League Against Rheumatism; HAQ = Health Assessment Questionnaire; anti-TNF = anti–tumor necrosis factor; RTX = rituximab.

† Propensity score included Disease Activity Score in 28 joints, comorbidities, the failed anti-TNF therapy, and interaction between age and reason for switching.

‡ Propensity score included age, comorbidities, the last failed anti-TNF therapy, interaction between disease duration and the reason for switching, using the logistic regression propensity score model.

### Physical function

The mean (95% CI) change in HAQ scores was similar between patients who switched to RTX (−0.13 [−0.17, −0.08]) and those who switched to a second anti-TNF therapy (−0.11 [−0.13, −0.08]) (Table 3). Within the 6 months of followup, 109 patients had either stopped or switched their therapy, and therefore were classified as non-MCID in HAQ achievers.

Patients who switched to RTX were more likely to achieve a MCID in HAQ score compared to those who switched to a second alternative anti-TNF therapy (*P* = 0.01). Of the patients who switched to RTX, 38.4% achieved a MCID in HAQ score compared to 29.6% of the patients who switched to a second anti-TNF therapy.

After adjustment for propensity scores, patients who switched to RTX were significantly more likely to achieve MCID improvements in HAQ score (OR 1.49 [95% CI 1.07, 2.08]) compared to those who switched to an alternative anti-TNF therapy (Table 4).

## DISCUSSION

This comparative effectiveness analysis addressed a clinically important question; which is more effective for RA patients who had failed their first anti-TNF therapy, switching to another alternative anti-TNF therapy or commencing RTX? Switching to RTX was found to be significantly more effective as measured by both clinical effectiveness (EULAR response) and patient-reported physical function (achieving a MCID in HAQ score) 6 months after switching.

To our knowledge, this is the first study to compare physical function after switching to either RTX or a second alternative anti-TNF therapy in RA patients who had failed their first anti-TNF therapy. A previous observational study by Finckh et al compared the change in DAS28 scores after switching to either RTX or alternative anti-TNF (12). However, the sample size was small: 50 patients switched to RTX and 66 patients switched to an alternative anti-TNF therapy compared to 387 and 941 patients, respectively, in the current analysis. A recent update of this study was of a larger sample size (155 and 163 patients, respectively) (20). However, the focus of this update was on subgroups of the patients according to the reason for switching or the number of failed anti-TNF therapies. The results of the current analysis come into agreement with the results of those previous observational studies that reported superior response in patients who switched to RTX (12, 20). This superior response to RTX may be explained by the new mechanism of action offered by RTX.

There was a higher rate of achieving disease remission in the patients who switched to an alternative anti-TNF therapy; however, it was not statistically significant. This might be due to the lower DAS28 score at the start of therapy in this group of patients. In the UK, retreatment with RTX is allowed no more frequently than every 6 months. Therefore, there is also the possibility that the DAS28 or HAQ scores recorded at 6 months were at the beginning of a disease flare. However, this was not supported by the overall finding of a better overall improvement for both outcomes in the RTX group at 6 months.

Previous analysis from the BSRBR had shown that, 6 months after initiating the first anti-TNF therapy in RA patients, the mean change in DAS28 score was −2.1 with 18% of the patients achieving a good EULAR response and 50% achieving a moderate response (21). In comparison to the results of the current analysis, it was noticed that the response to the second alternative anti-TNF therapy (mean change in DAS28 score −1.17, 13.5% good responders and 33.8% moderate responders) was relatively lower than that of the first anti-TNF therapy.

After failing the first anti-TNF therapy, the patients in both study arms were of similar physical function. The improvements in HAQ score 6 months after switching was small and did not reach the MCID in both treatments, which may suggest an irreversible physical disability in those patients who already have failed 1 anti-TNF therapy.

The BSRBR was established primarily to study the short- and long-term safety of biologic therapy, and as such was not developed to specifically address questions of treatment effectiveness. Nevertheless, the regular collection of DAS28 and HAQ scores allowed this secondary outcome to be analyzed. One limitation is that DAS28 and HAQ scores were not routinely collected at the time a subsequent biologic therapy was started, with the exception of those patients reregistered in the RTX cohort. Therefore, not all patients had a DAS28 and/or HAQ score recorded within the 3 months prior to the switch or at 6 months following the treatment switch, which explains the relatively lower proportion of patients included in these analyses compared to the total available. However, the very large sample size of the BSRBR had allowed running this analysis by matching the dates of switching, and the baseline or the followup data of the BSRBR resulted in a final sample size that is currently the largest to date addressing this specific clinical question.

On the other hand, based on the same limitation of not collecting a full “baseline” data set at a time of switching, the current analysis could not model the available data to study predictors of response to the second anti-TNF therapy. In addition, it was not recorded whether patients had experienced primary or secondary failure of their first anti-TNF therapy and, therefore, this question also cannot be addressed at this time. However, recently within the BSRBR, we have studied those factors associated with response to RTX (22). One important clinical question is whether patients who are rheumatoid factor (RF) negative should preferentially receive a second anti-TNF therapy over RTX. Interestingly, RF status was only a very weak predictor of change in DAS28 score and was not associated with EULAR response or DAS28 remission in our data set (22).

As the data were collected in the routine clinical setting, where clinic appointments may not fall precisely at timed intervals as per a strict study protocol, some allowances also needed to be made in terms of the timing of data collection. Therefore, we allowed a range of time in which to include study outcomes. As patients in the anti-TNF cohort would not have baseline data rerecorded at the time of the switch, baseline data on comorbidities were carried forward from the start of the first anti-TNF cohort for patients who switched to a second anti-TNF therapy (RTX switchers would have had this data recorded at the time of reregistering). It is possible that some patients may have developed new comorbidities during their first anti-TNF treatment time. However, a sample of 10% of the patients was checked (from the followup forms) to see whether new comorbidities had developed during this time, and 93% of these patients had not developed a new comorbidity.

Although there were more patients with ≥1 comorbidities in the arm of patients who switched to RTX rather than in those who switched to a second anti-TNF therapy, the response was higher in RTX patients. This may suggest that the comorbidity overall was not necessarily acting as a confounder. Unfortunately, low numbers within each type of comorbidity limited the ability to explore the role of any individual comorbidity as a potential confounder.

This analysis was limited to study the patients who had failed only 1 first anti-TNF therapy. In clinical practice, patients may switch their therapy after failing 1, 2, or even 3 anti-TNF therapies. Future analysis that considers multiple switchers is likely to be of interest, although sample sizes will be much smaller. Within those patients studied, multiple switching in the patients who switched to anti-TNF was noticed.

A limitation of this analysis might be that the anti-TNF patients were combined together (to maximize the power), which did not allow for the analysis of subgroups of patients separately. For instance, the proportions of patients switching to alternative treatments for a particular reason may differ in response from those switching for other reasons. However, our analysis allowed for adjustment for the reason for switching in the propensity models.

After the recent approvals of abatacept and tocilizumab, the options of available therapies to treat patients who have failed their first anti-TNF therapy increase. A future comparison of effectiveness after switching to abatacept, tocilizumab, RTX, or a second alternative anti-TNF therapy is likely to be of interest.

Finally, one of the limitations of this comparative analysis is inherited from the fact that this is an observational study and the patients were not randomized to switch to either RTX or a second alternative anti-TNF therapy. Therefore, there may have been certain characteristics that led physicians to choose one therapy over another. Some patients in this analysis would not have had the choice, as most of the anti-TNF switchers would have switched at a time that RTX was not available. However, to overcome this potential source of bias, the analyses were adjusted for propensity scores that considered differences in the baseline characteristics of the patients. A randomized controlled trial would help clarify some of these issues, but the importance of observational data in identifying these knowledge gaps is also recognized.

In conclusion, the results of this comparative analysis suggest that switching to RTX may be of more benefit than switching to a second alternative anti-TNF therapy in RA patients who have failed their first anti-TNF therapy. The benefits of RTX were superior in both achieving EULAR response and achieving a MCID in HAQ scores. These results suggest that, in clinical practice, for patients who had failed a first anti-TNF therapy, it may be better to start RTX at this point rather than switching to a second anti-TNF therapy. Future analysis that considers the response in multiple anti-TNF switchers, or specifically looking at the reason for the switching, is likely to be of interest.
